# Maternal starvation primes progeny response to nutritional stress

**DOI:** 10.1371/journal.pgen.1009932

**Published:** 2021-11-29

**Authors:** Kelly Voo, Jeralyn Wen Hui Ching, Joseph Wee Hao Lim, Seow Neng Chan, Amanda Yunn Ee Ng, Jasmine Yi Ying Heng, Shiao See Lim, Jun Wei Pek

**Affiliations:** 1 Singapore Polytechnic, Singapore, Singapore; 2 Ngee Ann Polytechnic, Singapore, Singapore; 3 Temasek Life Sciences Laboratory, National University of Singapore, Singapore, Singapore; 4 Department of Biological Sciences, National University of Singapore, Singapore, Singapore; 5 Raffles Institution, Singapore, Singapore; 6 Temasek Polytechnic, Singapore, Singapore; Sanford Burnham Prebys Medical Discovery Institute, UNITED STATES

## Abstract

Organisms adapt to environmental changes in order to survive. Mothers exposed to nutritional stresses can induce an adaptive response in their offspring. However, the molecular mechanisms behind such inheritable links are not clear. Here we report that in *Drosophila*, starvation of mothers primes the progeny against subsequent nutritional stress. We found that RpL10Ab represses TOR pathway activity by genetically interacting with TOR pathway components TSC2 and Rheb. In addition, starved mothers produce offspring with lower levels of RpL10Ab in the germline, which results in higher TOR pathway activity, conferring greater resistance to starvation-induced oocyte loss. The *RpL10Ab* locus encodes for the RpL10Ab mRNA and a stable intronic sequence RNA (*sisR-8*), which collectively repress RpL10Ab pre-mRNA splicing in a negative feedback mechanism. During starvation, an increase in maternally deposited RpL10Ab and *sisR-8* transcripts leads to the reduction of RpL10Ab expression in the offspring. Our study suggests that the maternally deposited RpL10Ab and *sisR-8* transcripts trigger a negative feedback loop that mediates intergenerational adaptation to nutritional stress as a starvation response.

## Author summary

In the wild, animals need to adapt to frequent changes in the environment. Mothers who are exposed to nutritional stresses are known to produce offspring which are preconditioned to adapt to the mothers’ environment. However, it is unclear how such maternal “memory” is being passed on to the offspring. Here we show that *Drosophila* mothers exposed to starvation produce offspring which are more resistant to starvation during oogenesis. This process is mediated by maternally inherited RpL10Ab mRNA and a stable intronic sequence RNA (*sisR-8*), which collectively repress the splicing of RpL10Ab pre-mRNA, leading to lower RpL10Ab expression in the offspring ovaries. As a consequence, lower RpL10Ab expression results in higher TOR pathway activity, conferring greater resistance to starvation during oogenesis. Hence, maternally inherited transcripts may play a role as mediators in conferring intergenerational adaption to starvation.

## Introduction

Studies in various organisms have suggested an adaptive function for maternal effects [1]. The egg contains abundant materials such as proteins, metabolites, organelles, mRNA, and noncoding RNA inherited from the mother that can potentially influence the fitness of the offspring [2]. Equally important is the effect of epigenetic modification of the oocyte DNA that can serve as a memory of maternal experience [2].

In *Caenorhabditis elegans (C*. *elegans)*, maternal environmental factors such as nutrient availability and osmotic conditions lead to changes in the maternal inheritance of small RNA, insulin signalling, vitellogenin and sugars, which protect the progeny against subsequent stressful conditions [3–7]. In human, starvation of the mothers had been proposed to lead to inappropriate adaptation, which subsequently resulted in a higher incidence of metabolic syndrome such as diabetes and obesity in the next generation [8]. However, the molecular mechanisms behind such an inheritable link are generally unknown.

Stable intronic sequence RNAs (sisRNAs) belong to a class of long noncoding RNAs that are stable and contain intronic sequences [9–11]. They can be produced in various ways such as canonical splicing and alternative splicing (via intron retention). They are also stabilized as linear and circular (or lariat) forms, and are able to regulate transcription, mRNAs and other noncoding RNAs [12–18]. In yeast, starvation or TOR inhibition leads to widespread increase in sisRNAs [19,20]. In *Drosophila*, starvation has been found to lead to an increase in the amount of maternally inherited sisRNA *sisR-2*, which regulates the homeostasis of primordial germ cells in the progeny [21]. Another maternally inherited sisRNA *sisR-4* has also been shown to influence the development of the offspring during embryogenesis in *Drosophila* [16]. Starvation leads to a dramatic decrease in stage 14 oocyte production, and it is unknown whether maternal starvation has any impact on offspring adaptation to starvation [22]. Thus, it is unknown whether maternally deposited sisRNAs may provide a molecular link between maternal starvation and the progeny’s adaptive response.

Although sisRNAs have been observed across diverse organisms, it remains unclear if they are generated from conserved genetic loci. Because some sisRNAs have been proposed to regulate their cognate genes in cis, finding sisRNAs from conserved genetic loci may suggest important auto-regulatory functions [14]. The RpL10A gene is under the control of an autoregulatory feedback loop, which is conserved in *C*. *elegans* and human [23]. The RpL10A protein binds to a conserved stretch of intron and inhibits splicing of the RpL10A pre-mRNA in a negative feedback mechanism. Interestingly, overexpression of RpL10Ab in *Drosophila* germline cells triggered apoptosis of stage 8/9 egg chambers, reminiscent of a starvation phenotype [24]. It is unknown whether the *RpL10Ab* locus in *Drosophila* generates any sisRNAs that are involved in the conserved autoregulatory feedback loop, or its potential involvement in the maternal transmission of starvation memory to the offspring.

Here we report that in *Drosophila*, starvation of mothers primes the progeny to better handle subsequent nutritional stress. The *RpL10Ab* locus encodes for the RpL10Ab mRNA and a sisRNA (*sisR-8*), which collectively repress RpL10Ab pre-mRNA splicing in a negative feedback mechanism. During starvation, an increase in maternally deposited RpL10Ab and *sisR-8* transcripts leads to reduction of RpL10Ab expression in the offspring. We found that RpL10Ab represses TOR pathway activity by genetically interacting with TOR pathway components TSC2 and Rheb. Lower levels of RpL10Ab in the progeny germline also lead to higher TOR pathway activity, which confers greater resistance to starvation-induced oocyte loss. Our study suggests that the maternally deposited RpL10Ab and *sisR-8* trigger a negative feedback loop that mediates intergenerational adaptation to nutritional stress as a starvation response.

## Results

### Identification of a sisRNA *sisR-8* from *Drosophila RpL10Ab* locus

**Fig 1:**
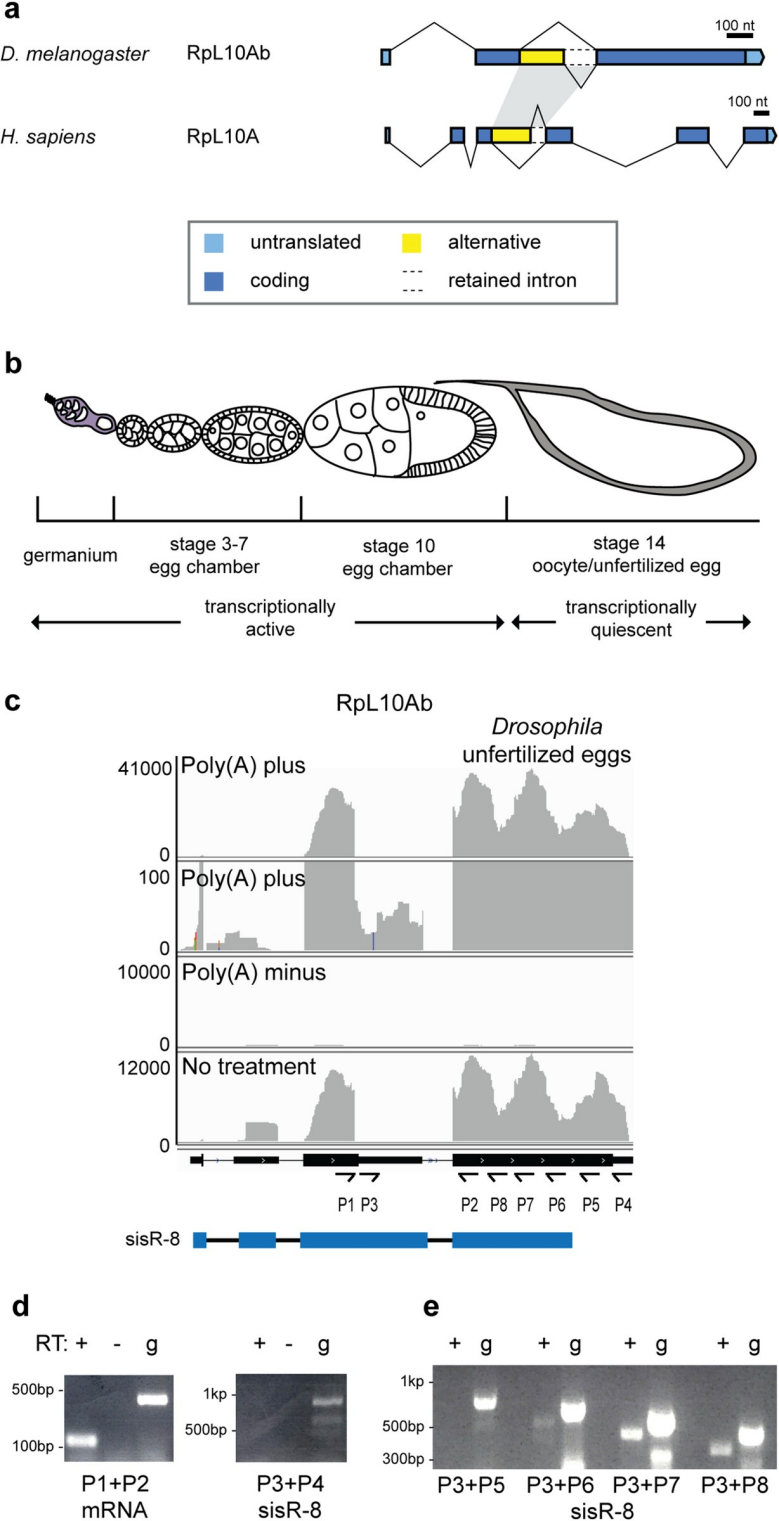
Identification of a sisRNA *sisR-8* from *Drosophila RpL10Ab* locus. (a) Exon-intron structures of RpL10A/RpL10Ab in fly (*D*. *melanogaster*) and human (*H*. *sapiens*). Gene annotations were retrieved from publicly available databases such as UCSC genome browser and FlyBase. Orthologous introns between the two species were highlighted in grey. (b) A drawing of an ovariole showing germline cells at different stages of oogenesis. To detect sisRNAs, unfertilized eggs were used as they are transcriptionally quiescent, hence no contamination of pre-mRNAs and unstable mRNAs that undergo nonsense mediate decay. (c) Genome browser view showing the presence of sisRNAs (reads mapping to intron 2) from RpL10Ab in *Drosophila* unfertilized eggs. Samples were enriched for poly(A) plus and minus RNA. An annotation of *sisR-8* derived from our experiments was shown in blue below. Arrows indicate the positions and directions of the primers used in D and E. (d, e) Agarose gels showing RT-PCR detecting the presence of *sisR-8* in *Drosophila* unfertilized eggs. A PCR product of the expected size was amplified using P1 + P2 showing the presence of RpL10Ab mRNA as a positive control. PCR products were not amplified using P3 + P4 and P3 + P5 although the primers can amplify bands of the expected sizes using genomic DNA as positive controls. PCR products of the expected size was amplified using P3 + P6, P3 + P7 and P3 + P8, consistent with *sisR-8* having a shorter 3’ end than the mRNA. In (E) RT+ bands were shorter than those from gDNA due to splicing, excluding gDNA contamination. g = genomic DNA as positive controls.

To search for potentially conserved sisRNA from the *Drosophila RpL10Ab* locus, we compared the gene structures of human and *Drosophila* RpL10Ab (Fig 1A). Previously, *Drosophila RpL10Ab* intron 2 was reported to be orthologous to human *RpL10Ab* intron 3. A short stretch of 40 nucleotides within the orthologous intron (Fig 1A, yellow) is highly conserved from *C*. *elegans* to human. Furthermore, a portion of the orthologous intron (Fig 1A, yellow) can be included as an exon by alternative splicing using a downstream 5’ splice site. Taken together, these observations suggest that a portion of intron 2 may be retained as a sisRNA via alternative splicing.

To determine whether intron 2 of *Drosophila* RpL10Ab produces a sisRNA, we examined previously published RNA sequencing data from unfertilized eggs [12]. The unfertilized egg is an excellent system to unambiguously identify sisRNAs because it is transcriptionally quiescent and contains a pool of stable RNA (Fig 1B). Reads mapping to the alternatively spliced intron 2 were detected in poly(A)-plus but not poly(A)-minus samples, suggesting the presence of polyadenylated sisRNA (Fig 1C). To confirm this, we performed RT-PCR on RNA from unfertilized eggs. As a positive control, primers P1 and P2 detected the presence of RpL10Ab mRNA. However, using primers P3 (specific to intron 2) and P4 (complementary to the extreme 3’ end of RpL10Ab mRNA), we did not detect any PCR product, despite the fact that they can amplify a product from genomic DNA (Fig 1D).

To verify the length of the sisRNA, we designed reverse primers that span the 3’ end of the transcript (P5-8). All of the reverse primers (except P5) were able to amplify a PCR product with P3, confirming that sisR-8 has a shorter 3’ end compared to RpL10Ab mRNA (Fig 1C, blue, and 1E).

### Autoregulation of RpL10Ab expression by RpL10Ab protein and *sisR-8*

**Fig 2:**
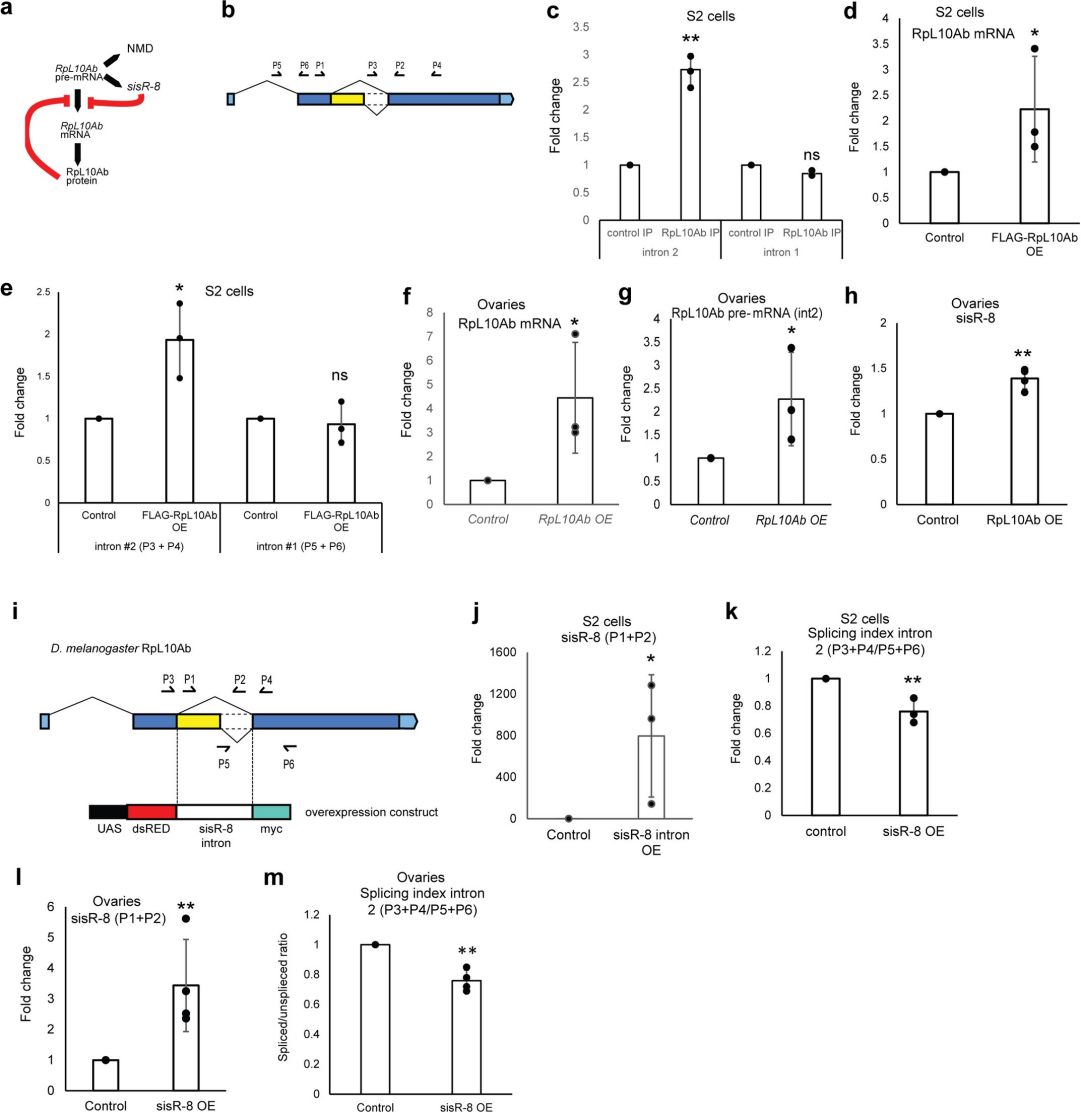
Autoregulation of RpL10Ab by RpL10Ab protein and *sisR-8*. (a) Model showing the negative feedback loop mediated by *sisR-8* and RpL10Ab protein. Both *sisR-8* and RpL10Ab protein repress splicing of RpL10Ab intron 2, which leads to increase in both *sisR-8* and an unstable transcript degraded by NMD. (b) Genetic locus of RpL10Ab. Arrows indicate the positions and directions of the primers used. (c) Relative levels of intron 1 and 2 in control and RpL10Ab IP as measured by qRT-PCR. Error bars depict SD from n = 3 biological replicates. **: p<0.01. ns: p>0.05. (d, e) Relative levels of RpL10Ab mRNAs (P1 + P2) and pre-mRNA (respective primers shown in figure) in control versus RpL10Ab overexpression S2 cells as measured by qRT-PCR. Error bars depict SD from n = 3 biological replicates. *: p<0.05. ns: p>0.05. (f, g) Relative levels of RpL10Ab mRNAs (P1 + P2) and pre-mRNA (respective primers shown in figure) in control versus RpL10Ab overexpression ovaries as measured by qRT-PCR. Error bars depict SD from n = 3 biological replicates. (h) Relative levels of *sisR-8* in control versus RpL10Ab overexpression ovaries as measured by qRT-PCR. Error bars depict SD from n = 3 biological replicates. **: p<0.01. (i) Genetic locus for *Drosophila* RpL10Ab. Arrows indicate the positions and directions of the primers used. Intron 2 is being cloned between dsRed and myc for overexpression assay. (j) Relative levels of *sisR-8* in control versus *sisR-8* intron overexpression S2 cells as measured by qRT-PCR. Error bars depict SD from n = 3 biological replicates. *: p<0.05. (k) Splicing efficiency (spliced/unspliced ratio) in control versus *sisR-8* intron overexpression S2 cells as measured by qRT-PCR. Error bars depict SD from n = 3 biological replicates. **: p<0.01. (l) Relative levels of *sisR-8* in control versus *sisR-8* intron overexpression ovaries as measured by qRT-PCR. Error bars depict SD from n = 4 biological replicates. **: p<0.01. (m) Splicing efficiency (spliced/unspliced ratio) in control versus *sisR-8* intron overexpression ovaries as measured by qRT-PCR. Error bars depict SD from n = 4 biological replicates. **: p<0.01. All qRT-PCR were normalized against *actin5C* mRNA.

In *C*. *elegans* and human, the splicing of intron 2 had been shown to be regulated by the RpL10Ab protein, suggesting a conserved regulatory feedback loop between the two [23]. Furthermore, other sisRNAs have been shown to engage in autoregulatory feedback loops [14]. We therefore examined whether the splicing of *Drosophila RpL10Ab* intron 2 is also regulated by RpL10Ab and *sisR-8* (Fig 2A). Recombinant RpL10A protein binds to the orthologous *RpL10Ab* intron 2 in vitro [23]. Using *Drosophila* S2 cell lysates, we confirmed that *Drosophila* RpL10Ab binds to intron 2 but not intron 1 (Fig 2B and 2C). Consistent with studies done in *C*. *elegans* and human, overexpression of RpL10Ab in S2 cells also resulted in the inhibition of intron 2 splicing but not intron 1, as indicated by the upregulation of unspliced transcripts containing intron 2 (Fig 2D and 2E). To show this effect in vivo, we generated transgenic flies overexpressing RpL10Ab under the control of the germline-specific *MTD-Gal4* driver (Fig 2F). Similar to results obtained from S2 cells, we observed a decrease in intron 2 splicing as indicated by the increase in intron 2 containing pre-mRNA (Fig 2G). Accordingly, we also observed a modest increase in *sisR-8* levels, supporting the idea that inhibition of RpL10Ab pre-mRNA splicing leads to higher production of *sisR-8* (Fig 2H).

We next overexpressed the *sisR-8* intron by cloning *RpL10Ab* intron 2 into the UAS-dsRed-intron-myc construct (referred to as *sisR-8* overexpression) (Fig 2I). *sisR-8* overexpression resulted in the down-regulation of intron 2 splicing as indicated by the drop in splicing index (mRNA/pre-mRNA) (Fig 2J and 2K). In vivo, a similar decrease in splicing index was also observed in the germline cells of transgenic flies overexpressing *sisR-8* (Fig 2I and 2M). Taken together, our experiments are consistent with the model that increase in both RpL10Ab and *sisR-8* suppress splicing of RpL10Ab pre-mRNA (Fig 2A).

### A memory of maternal starvation on offspring

**Fig 3:**
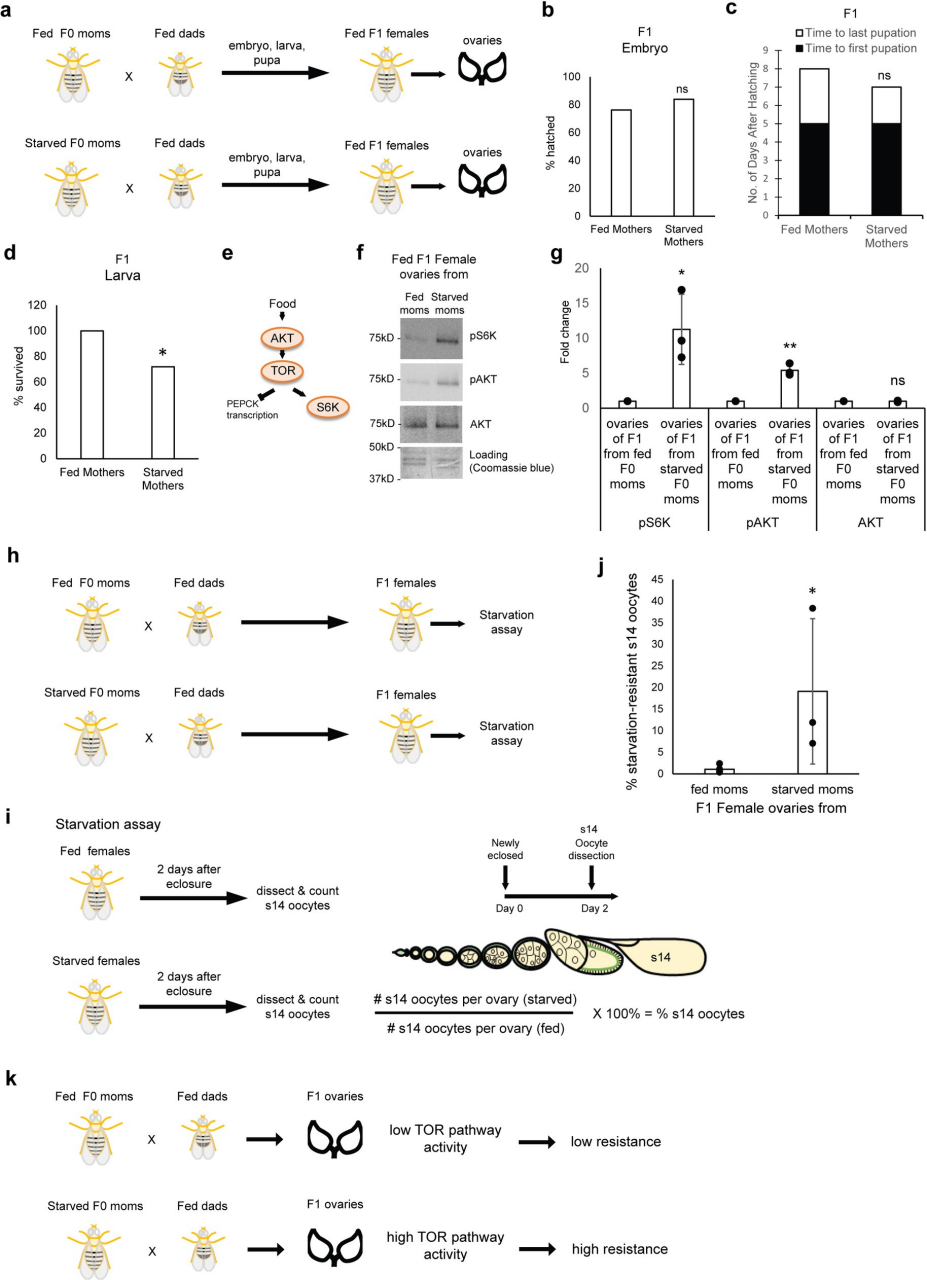
A memory of maternal starvation on offspring. (a) Experimental scheme used to study the effects of maternal starvation on the offspring. Newly eclosed fed or starved F0 mothers were mated with fed fathers for 2 days. The offspring embryonic, larval and pupal developments were monitored. Newly eclosed F1 females were fatten up (fed) for 2 days before ovaries were dissected for western blot analyses. (b) Hatch rates of F1 embryos from fed and starved F0 mothers. ns: p>0.05, Fischer exact test. (c) Number of days after hatching to first and last pupation of F1 larvae from fed and starved F0 mothers. ns: p>0.05, Fischer exact test. (d) Survival rates of F1 larvae to pupation from fed and starved F0 mothers. *: p<0.001, Fischer exact test. (e) Diagram showing the components of the TOR pathway assayed in the experiments. Food induces TOR pathway activity by activating AKT, TOR and S6K, but represses the transcription of PEPCK. (f) Western blots showing the amounts of pS6K, pAKT and AKT proteins in ovaries from fed F1 progeny from starved and fed F0 mothers (shown in A). Coomassie blue staining of gel was used a loading control. Proteins equivalent to the same number of ovaries were loaded in each well. (g) Quantification of the western blots shown in (g). n = 3 biological replicates. *p<0.05; **: p<0.01. ns: p>0.05.(h, i) Diagrams showing the experimental scheme to assay for the starvation-resistance of stage 14 oocytes in the F1 offspring from fed or starved F0 mothers. (h) Fed or starved F0 mothers were mated with fed fathers. The F1 females were immediately exposed to the starvation assay after eclosure. (i) To perform the starvation assay, F1 females were either fed or starved for 2 days immediately after eclosure. They were then dissected to quantify the number of stage 14 oocytes in each condition. Percentage of starvation-resistant stage 14 oocytes was calculated using the formula shown. (j) % of starvation-resistant stage 14 oocytes of F1 progeny from starved F0 mothers compared to fed F0 mothers. *: p<0.05. (k) Diagram summarizing the results shown in this figure. F1 progeny ovaries from starved F0 mothers have a higher level of TOR pathway activity, which makes them more resistant to starvation.

What could be the biological significance of the RpL10Ab negative feedback loop? One possibility is its roles in transmitting the memory of starvation from the mother to the offspring. To examine this hypothesis, we crossed fed and starved wildtype F0 mothers to fed fathers and observed the development of the F1 embryos, larvae and pupae. We also fed the F1 females for 2 days (immediately after eclosure) before dissecting their ovaries for analyses (Fig 3A). While F1 offspring from both fed and starved F0 mothers have similar rates of embryonic, larval and pupal development (Fig 3B and 3C), we observed that F1 larvae from starved F0 mothers had a lower survival rate (~70% of larvae survived to pupation from starved F0 mothers vs 100% from fed F0 mothers) (Fig 3D). To examine the memory of starvation, we assayed the activity of the TOR pathway in the F1 offspring of fed and starved F0 mothers. Compared to F1 offspring from fed F0 mothers, the ovaries of fed F1 offspring from starved F0 mothers showed higher pS6K and pAKT levels, indicating a higher TOR pathway activity (Fig 3E, 3F and 3G).

During starvation, stage 14 oocytes production is dramatically reduced due to apoptosis of stage 8/9 egg chambers [22]. This is in part due to downregulation of TOR pathway activity [25–27]. Thus, we reasoned that ovaries with higher levels of TOR pathway activity should be more resistant to starvation-induced oocyte loss. To measure starvation-induced oocyte loss in the F1 offspring, newly eclosed female offspring from both fed and starved F0 mothers were starved or fed for 2 days before dissection to quantify the number of stage 14 oocytes in each ovary (Fig 3H and 3I). The percentage of starvation-resistant stage 14 oocytes was then calculated by dividing the number of stage 14 oocytes per ovary (starved) by the number of stage 14 oocytes per ovary (fed) (see Materials and Methods) (Fig 3I). The F1 offspring with a higher percentage of starvation-resistant stage 14 oocytes was interpreted as having a greater resistance to starvation-induced oocyte loss. F1 offspring from fed F0 mothers consistently displayed low (~1%) percentages of starvation-resistant stage 14 oocytes (Fig 3J), in agreement with the dramatic decrease in oogenesis during starvation as previously reported [22]. In line with a higher TOR pathway activity, F1 offspring from starved F0 mothers generally had a higher percentage of starvation-resistant stage 14 oocytes ranging from ~6–35% (Fig 3J). Note that the variance is caused by batch-to-batch variation as we performed each biological replicates on different days to ensure that similar observations can be seen. Although the there is a variance from 6–35%, the trend is the same in all biological replicates where the percentage of starvation-resistant oocytes increases. Taken together, we conclude that F1 offspring from starved F0 mothers retain a memory that upregulates TOR pathway activity in the F1 ovaries, conferring them greater resistance to starvation-induced oocyte loss (Fig 3K).

### Maternally deposited RpL10Ab and *sisR-8* are required for transmission of starvation memory

**Fig 4:**
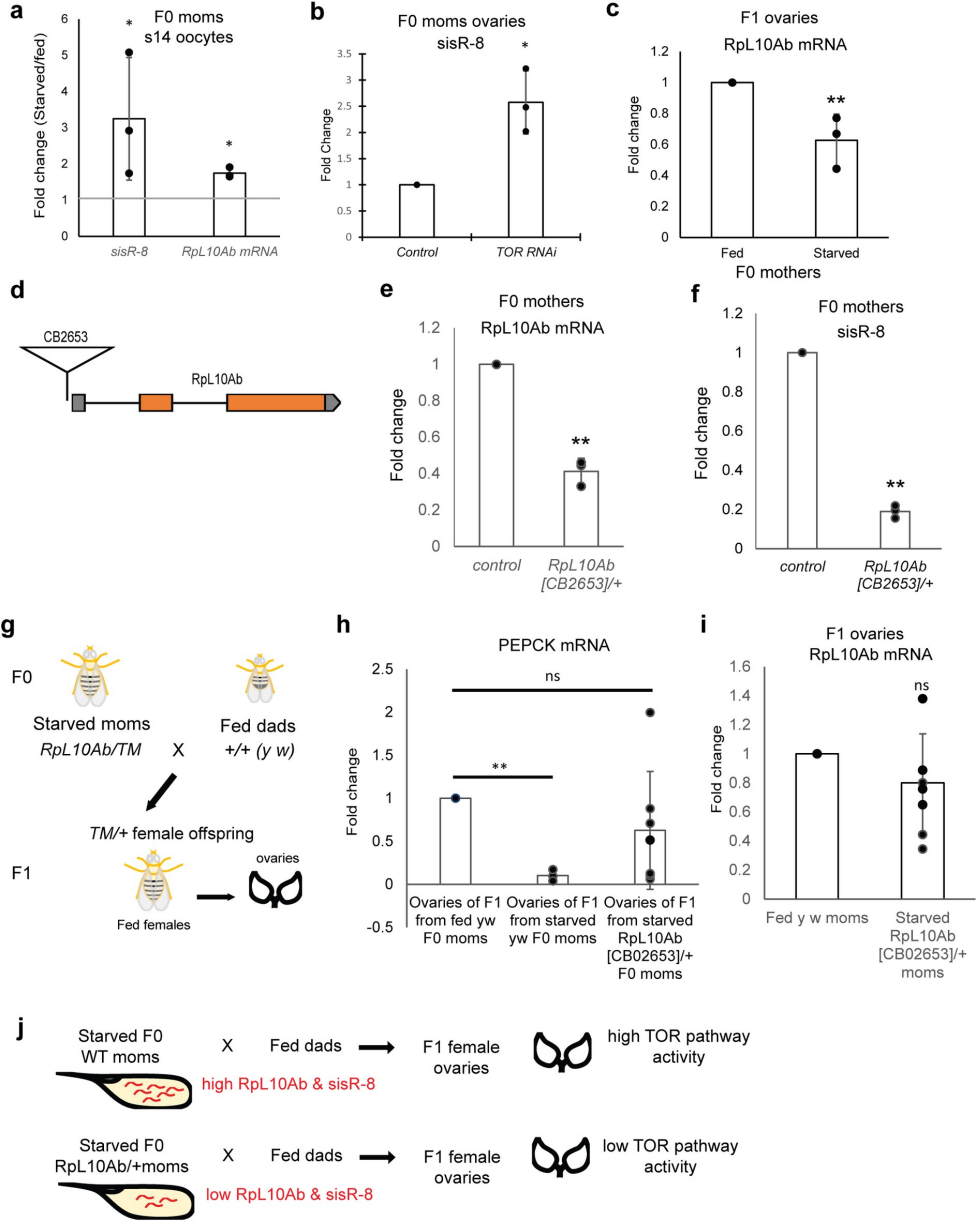
Maternally deposited RpL10Ab and *sisR-8* are required for transmission of starvation memory. (a) Fold-change of *sisR-8* and RpL10Ab in stage 14 oocytes from starved compared to fed F0 mothers. Starvation induces higher levels of *sisR-8* and RpL10Ab mRNA in stage 14 oocytes. *: p<0.05, t-test. Error bars represent sd from mean from 3 biological replicates. (b) Fold-change of *sisR-8* in *MTD-Gal4>TOR* RNAi compared to control ovaries. *: p<0.01, t-test. Error bars represent sd from mean from 3 biological replicates. (c) Fold-change of RpL10Ab mRNA in ovaries from fed F1 progeny from starved F0 mothers compared to those from fed F0 mothers (refer to Fig 3A). Error bars represent sd from mean from 3 biological replicates. **: p<0.01. (d) Gene structure of RpL10Ab showing the insertion CB02653 at the promoter region. (e, f) Insertion at the RpL10Ab promoter disrupts both RpL10Ab and *sisR-8* expression. Fold-change of *sisR-8* and RpL10Ab in ovaries from fed *RpL10Ab[CB02653]/+* compared to fed control F0 mothers. Error bars represent sd from mean from 3 biological replicates. **: p<0.01. (g) Crossing scheme to reduce a copy of *RpL10Ab* gene in the F0 mothers and the collection of *TM/+* F1 offspring. (h) Fold-change of PEPCK in F1 offspring ovaries from starved wildtype or starved *RpL10Ab[CB2653]/+* F0 mothers compared to those from fed wildtype F0 mothers. Error bars represent sd from mean from 3–7 biological replicates. **: p<0.01, ns: p>0.05. (i) Fold-change of RpL10Ab mRNA in fed F1 offspring ovaries from starved *RpL10Ab[CB2653]/+* F0 mothers compared to those from fed wildtype F0 mothers. Error bars represent sd from mean from 7 biological replicates. ns: p>0.05. All qRT-PCR were normalized against *actin5C* mRNA. (j) Diagram summarizing the results shown in this figure. Maternally deposited RpL10Ab and *sisR-8* mediate the transmission of maternal starvation memory to the offspring.

To investigate if RpL10Ab and *sisR-8* mediated regulation of RpL10Ab expression is required for the inheritance of starvation memory from mothers, we examined the levels of maternally deposited RpL10Ab and *sisR-8* transcripts. Newly eclosed F0 female flies were starved or fed for 2 days before dissecting the stage 14 oocytes for RNA extraction (Fig 3l). Interestingly, both RpL10Ab and *sisR-8* were up-regulated in the stage 14 oocytes of starved F0 flies (Fig 4A). As a positive control for starvation, PEPCK was also upregulated (S1A Fig). It was previously shown that TOR pathway inactivation led to the upregulation of sisRNAs in yeast [19]. Similarly, we observed an increase in *sisR-8* levels in TOR RNAi ovaries (Figs 4B and S1B). Consistent with the observations that increase in RpL10Ab and *sisR-8* levels leads to the downregulation of RpL10Ab expression (Fig 2), we observed a downregulation of RpL10Ab mRNA expression in the ovaries of fed F1 offspring from starved F0 mothers compared to fed F0 mothers (Fig 4C). This observation suggests that up-regulation of both maternally deposited RpL10Ab and *sisR-8* is linked to down-regulation of RpL10Ab mRNA in the fed F1 offspring ovaries.

To test whether maternal RpL10Ab and *sisR-8* are mediators of the intergenerational starvation memory, we used a promoter insertion mutant that disrupts the transcription of RpL10Ab and *sisR-8* (Fig 4D). As homozygous *RpL10Ab[CB02653]* mutants are lethal, we used heterozygous mutants that have reduced levels of RpL10Ab and *sisR-8* expression (Fig 4E and 4F). We crossed starved *RpL10Ab[CB02653]/+* F0 mothers to fed wildtype fathers and selected *TM/+* female F1 offspring for further analysis (Fig 4G).

As the number of offspring produced in this intergenerational starvation assay is severely limited, experimental designs targeting our protein of interests were not possible. To overcome this, we collected ovary pairs from a single F1 offspring for quantitative and sensitive qPCR instead. PEPCK (a downstream target of TOR pathway) was used as a more sensitive readout for TOR pathway activity (Fig 3E). As expected, ovaries of F1 offspring from starved wildtype F0 mothers have significantly lower levels of PEPCK compared to those from fed F0 mothers due to higher TOR pathway activity (Fig 4H). However, in some F1 offspring from starved *RpL10Ab[CB02653]/+* F0 mothers, PEPCK levels did not decrease (Fig 4H), suggesting that TOR pathway activity was not consistently upregulated. Importantly, the expression of RpL10Ab was not consistently downregulated in F1 offspring ovaries from starved *RpL10Ab[CB02653]/+* F0 mothers as well (Fig 4I compare to Fig 4C). The highly divergent distribution of the expression of PEPCK and RpL10Ab mRNA in F1 offspring ovaries from starved *RpL10Ab[CB02653]/+* F0 mothers suggests that their expression is not being robustly repressed. We hypothesize that there may be additional pathways or mechanisms that govern this robustness and lowering the amounts of maternally deposited RpL10Ab and *sisR-8* exposes the F1 offspring to such ‘hidden’ variation in gene expression. As a control, we performed the reciprocal cross and observed that the F1 offspring still retained the memory as indicated by a consistent down-regulation of PEPCK and RpL10Ab expression (S2 Fig). Thus, we conclude that maternally deposited RpL10Ab and *sisR-8* are required for intergenerational transmission of starvation memory by downregulating RpL10Ab expression in the F1 offspring (Fig 4J).

### RpL10Ab suppresses TOR pathway activity

**Fig 5:**
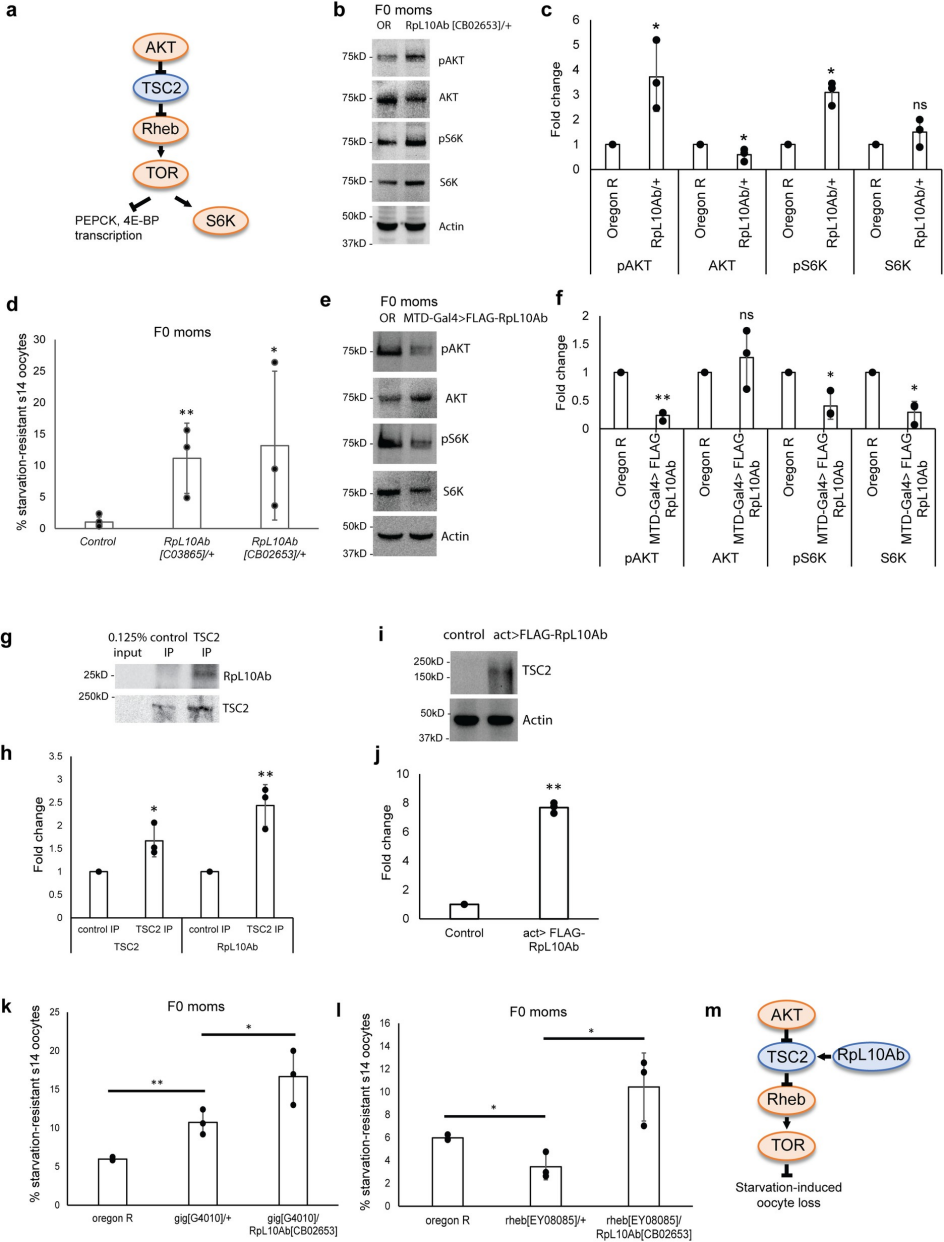
RpL10Ab suppresses TOR pathway activity. (a) Diagram showing the components of the TOR pathway assayed in the experiments. Orange and blue represent positive and negative regulators of TOR pathway respectively. (b) Western blots showing the amounts of pS6K, pAKT, S6K, AKT and Actin proteins in ovaries from fed *Oregon R* and *RpL10Ab[CB02653]/+* F0 flies. (c) Quantification of the western blots shown in (b). n = 3 biological replicates. *p<0.05. ns: p>0.05. (d) % of starvation-resistant stage 14 oocytes in *RpL10Ab[C03865]/+* and *RpL10Ab[CB02653]/+* F0 mothers compared to F0 control. *: p<0.05, **: p<0.01. (e) Western blots showing the amounts of pS6K, pAKT, S6K, AKT and Actin proteins in ovaries from fed *Oregon R* and *MTD-Gal4>FLAG-RpL10Ab* F0 flies. (f) Quantification of the western blots shown in (e). n = 3 biological replicates. *p<0.05. **p<0.01. ns: p>0.05. (g) Western blots showing the co-immunoprecipitation between TSC2 and RpL10Ab in S2 cells. (h) Quantification of the western blots shown in (g). n = 3 biological replicates. *p<0.05. **p<0.01. (I) Western blots showing the levels of TSC2 protein in control versus S2 cells overexpressing FLAG-RpL10Ab. (j) Quantification of the western blots shown in (I). n = 3 biological replicates. **p<0.01. (k, l) % of starvation-resistant stage 14 oocytes in the indicated genotypes, indicating a genetic interaction between RpL10Ab and *gig* and *rheb*. *: p<0.05, **: p<0.01. (m) A genetic pathway showing the involvement of RpL10Ab in the TOR pathway in the regulation of starvation-induced stage 14 oocyte loss.

Our results suggest that maternal starvation results in higher levels of maternally deposited RpL10Ab and *sisR-8*, which repress RpL10Ab expression in the F1 offspring. This in turn leads to higher TOR pathway activity in the F1 ovaries, which reduces starvation-induced oocyte loss in the F1 offspring. Overexpression of RpL10Ab in germline cells led to apoptosis, which is reminiscent of the starvation response [24]. To examine whether RpL10Ab regulates TOR pathway activity, we assayed some relevant components of the TOR pathway (Fig 5A). In *RpL10Ab/+* F0 ovaries, pAKT and pS6K were upregulated while the expression of PEPCK and 4E-BP mRNAs was downregulated (Figs 5B, 5C, S3A, S3B, S4A and S4B), indicative of a higher TOR pathway activity. Two *RpL10Ab/+* alleles were also more resistant to starvation-induced oocyte loss (Figs 5D, S3C, and S3D). To confirm that RpL10Ab regulates TOR pathway activity, we overexpressed RpL10Ab in the ovaries using *MTD-Gal4* driver and observed a downregulation of pS6K and pAKT, along with the upregulation of PEPCK (Figs 5E, 5F, S3E, S4C and S4D), indicative of a reduction in the TOR pathway activity. Taken together, our experiments suggest that RpL10Ab suppresses TOR pathway activity.

To further characterize how RpL10Ab modulates the TOR pathway, we performed physical and genetic interaction analyses of RpL10Ab with known components of the TOR pathway. On FlyBase, RpL10Ab has been shown to bind to TSC2 (or *gig*), a negative regulator of TOR pathway by repressing Rheb (Fig 5A). We confirmed an in vivo interaction between TSC2 and RpL10Ab in S2 cells by co-immunoprecipitation (Fig 5G and 5H). Furthermore, overexpression of RpL10Ab led to a higher abundance of TSC2 protein (Fig 5I and 5J), suggesting that RpL10Ab binds to and promotes TSC2 protein abundance, possibly by promoting its stability.

We next investigate whether RpL10Ab genetically interacts with TSC2 in the regulation of starvation-induced oocyte loss. TSC2 heterozygous mutants (*gig/+*) had a higher percentage of starvation-resistant stage 14 oocytes (or more resistant to starvation-induced oocyte loss) (Fig 5K), consistent with the idea that TOR pathway activity suppresses starvation-induced oocyte loss. Moreover, reducing a copy of RpL10Ab enhances the *TSC2/+* phenotype (Fig 5K). Next, we examined Rheb, which is a positive regulator of TOR pathway) (Fig 5A). *Rheb/+* heterozygous mutants were more sensitive to starvation-induced oocyte loss as indicated by a decrease in percentage of starvation-resistant stage 14 oocytes (Fig 5l). This observation further supports the idea that the TOR pathway suppresses starvation-induced oocyte loss. Consistent with the idea that RpL10Ab promotes TOR pathway activity, reducing a copy of RpL10Ab suppressed the *rheb/+* phenotype (Fig 5l). Together, our experiments provide evidence for the role of RpL10Ab in repressing the TOR pathway activity in the regulation of starvation-induced oocyte loss (Fig 5M).

It was previously proposed that the TOR pathway acts to regulate the apoptosis of stage 8/9 egg chambers during nutritional stress [27]. During starvation, TOR pathway is downregulated, and this leads to increase in apoptosis of stage 8/9 egg chambers and a consequent reduction in stage 14 oocyte production. We examined the percentages of stage 8/9 egg chambers undergoing apoptosis in starved *Oregon R*, *RpL10Ab/+* and *gig/+* heterozygous mothers by staining with an antibody against cleaved *Drosophila* Dcp-1 protein. Apoptotic stage 8/9 egg chambers were stained positive for Dcp-1 protein (S5A Fig, arrowheads). The results showed a significant decrease in the percentages of apoptotic stage 8/9 egg chambers in both *RpL10Ab/+* and *gig/+* heterozygous mothers compared to *Oregon R* mothers (S5B Fig, from ~12.6% in *Oregon R* to ~1.6% in *RpL10Ab/+* and ~2.3% in *gig/+*). Our results corroborate the idea that RpL10Ab and the TOR pathway act through a common upstream event (via apoptosis) to regulate oocyte production.

### *sisR-1* to *sisR-4* are upregulated during starvation and promote stage 14 oocyte loss

**Fig 6:**
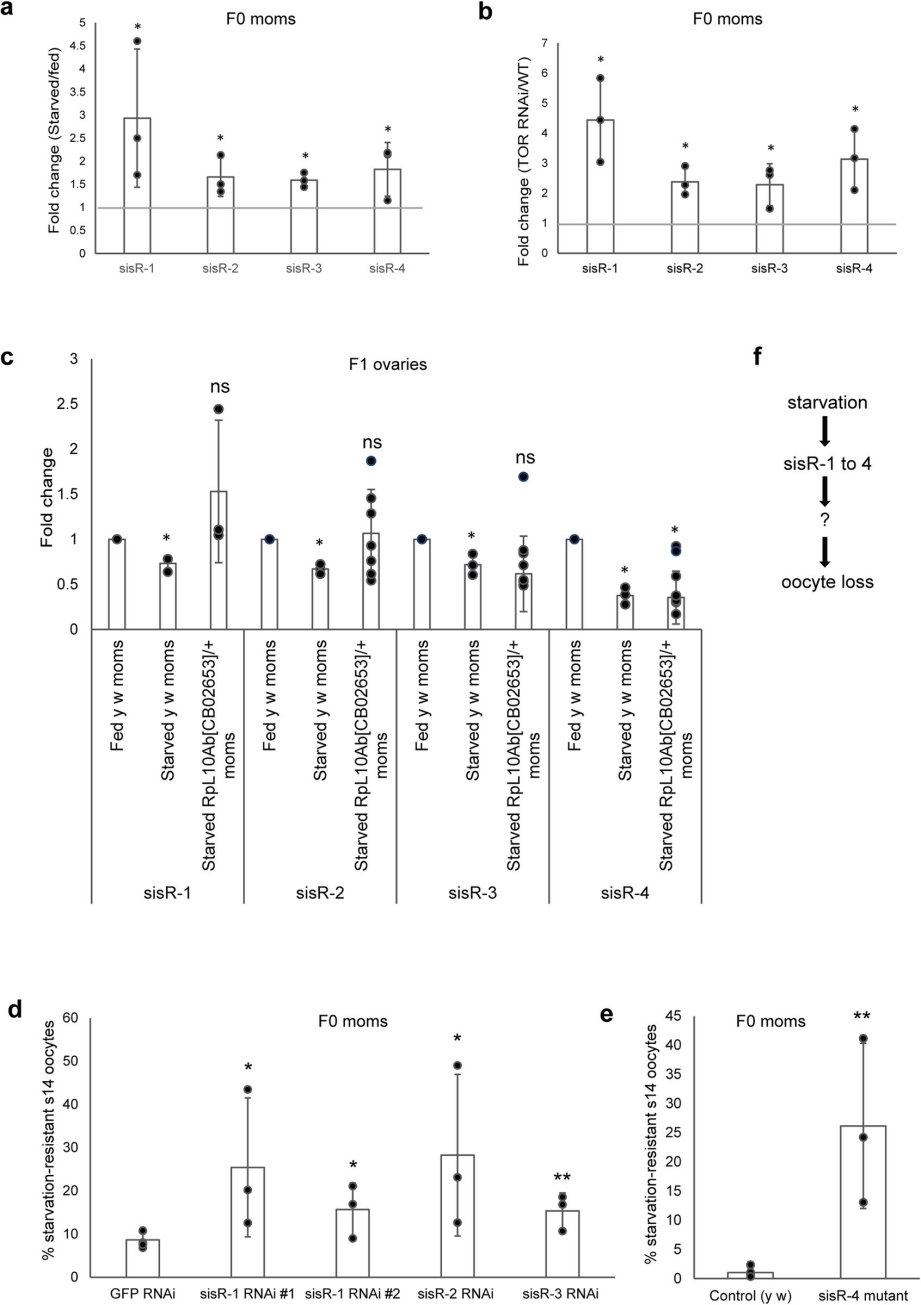
*sisR-1* to *sisR-4* are upregulated during starvation and promote stage 14 oocyte loss. (a) Fold-change of *sisR-1* to *sisR-4* in stage 14 oocytes from starved F0 mothers compared to fed F0 mothers. *: p<0.05, t-test. Error bars represent sd from mean from 3 biological replicates. (b) Fold-change of *sisR-1* to *sisR-4* in *MTD-Gal4>TOR* RNAi compared to control ovaries. *: p<0.01, t-test. Error bars represent sd from mean from 3 biological replicates. (c) Fold-change of *sisR-1* to *sisR-4* in F1 offspring ovaries from starved wildtype or starved *RpL10Ab[CB2653]/+* F0 mothers compared to those from fed wildtype F0 mothers. Error bars represent sd from mean from 3–7 biological replicates. *: p<0.01, ns: p>0.05. All qRT-PCR were normalized against *actin5C* mRNA. (d, e) % of starvation-resistant stage 14 oocytes in the indicated genotypes. *: p<0.05, **: p<0.01. (f) Diagram summarizing the results shown in this figure. Starvation induces the expression of *sisR-1* to *sisR-4*, which lead to oocyte loss via an unknown mechanism.

*sisR-8*, together with *sisR-2*, are upregulated in starved stage 14 oocytes (Fig 4A) [21]. In yeast, starvation leads to general upregulation of sisRNAs [19,20]. We wondered whether other previously identified *Drosophila* sisRNAs are also regulated by starvation. Indeed, *sisR-1* to *sisR-4* were upregulated in starved stage 14 oocytes (Fig 6A) [12,13,15,16]. These results were confirmed in TOR RNAi ovaries (Fig 6B), indicating that TOR pathway suppresses sisRNA production. Consistent with the upregulation of TOR activity in the ovaries of F1 offspring from starved F0 mothers, we found that the levels of *sisR-1* to *sisR-4* were downregulated (Fig 6C). In addition, the suppression of *sisR-1* to *sisR-4* was alleviated in the ovaries of offspring from starved *RpL10Ab/+* F0 mothers (Fig 6C).

Since *sisR-1* to *sisR-4* are all upregulated in starved ovaries, we examined if they may play a role in regulating starvation-induced oocyte loss. Using previously published RNAi lines that knock down *sisR-1* to *sisR-3* and a *sisR-4* mutant, we examined the percentages of stage 14 oocytes remaining after starvation (Fig 6D) [12,13,15,16]. Using GFP RNAi as control, which exhibited a consistently low percentage of starvation-resistant stage 14 oocytes, the RNAi of *sisR-1* to *sisR-3* resulted in a general increase in the percentage of starvation-resistant stage 14 oocytes (Fig 6D). In addition, *sisR-4* mutants also exhibited a higher percentage of starvation-resistant stage 14 oocytes compared to the control (Fig 6E). Thus, our results suggest that the upregulation of four sisRNAs during starvation may play a role in making the ovaries more sensitive to starvation-induced oocyte loss (Fig 6F).

## Discussion

**Fig 7:**
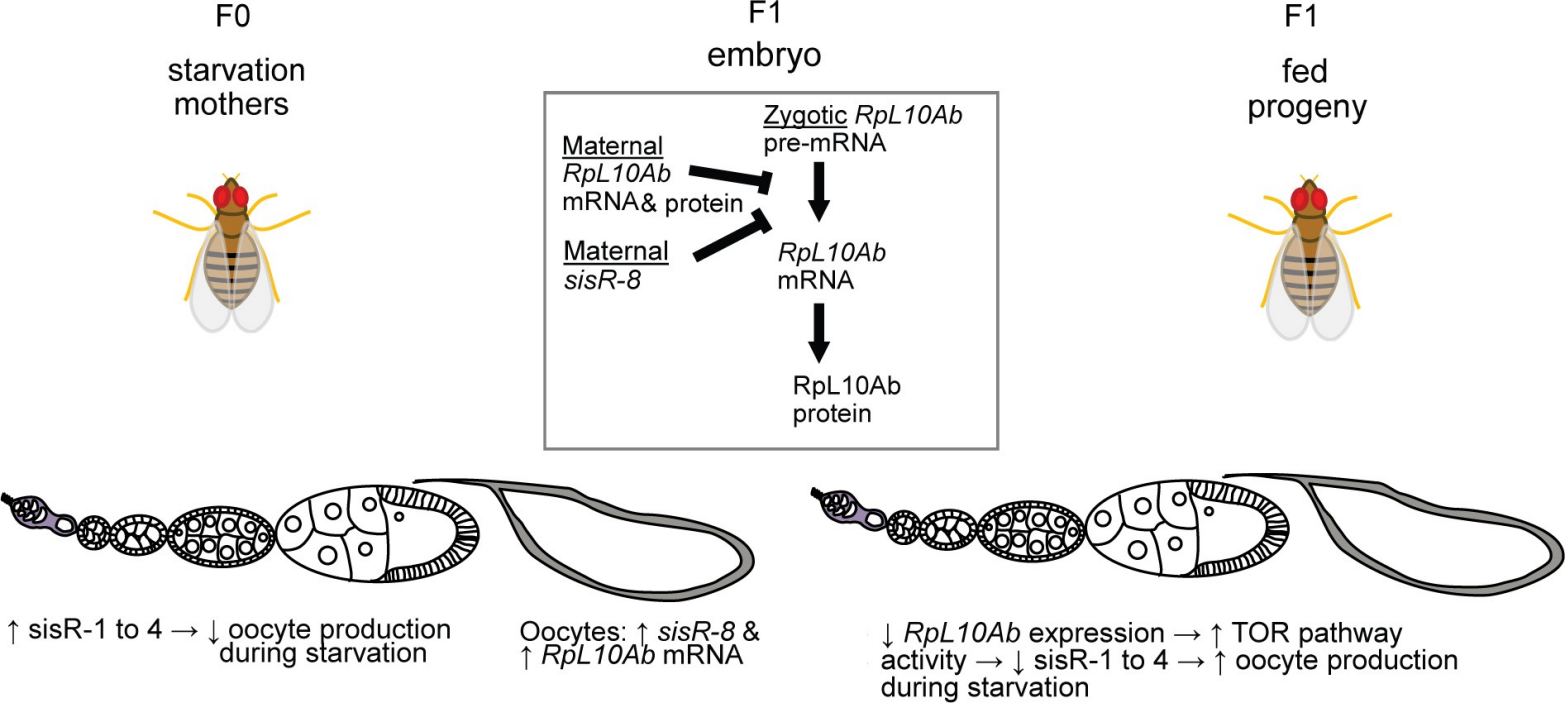
Model showing the intergenerational inheritance of starvation memory from the mothers to the progeny via the RpL10Ab negative feedback loop. Starvation increases the expression of four sisRNAs (*sisR-1* to *sisR-4*) in the ovaries, which results in a decrease in oocyte production. Those oocytes that managed to develop past the apoptotic checkpoint have higher levels of *sisR-8* and RpL10Ab mRNA, which is later translated into RpL10Ab protein during development. Both *sisR-8* and RpL10Ab repress the splicing of RpL10Ab pre-mRNA transcribed during development and result in a lower basal expression of RpL10Ab. This leads to a more active TOR pathway in the progeny germline, lower *sisR-1* to *sisR-4* expression and consequently greater resistance to starvation-induced oocyte loss.

In this study, we propose that during starvation, oocytes that managed to develop past the stage 8/9 apoptotic checkpoint have higher levels of *sisR-8* and RpL10Ab mRNA, which is later translated into RpL10Ab protein during development. During development, both *sisR-8* and RpL10Ab repress the splicing of RpL10Ab pre-mRNA lowering the basal expression of RpL10Ab in the F1 ovaries. In the progeny germline, lower RpL10Ab leads to a more active TOR pathway, in turn reducing *sisR-1* to *sisR-4* expression and consequently provide greater resistance to starvation-induced oocyte loss (Fig 7).

The regulation of offspring response to starvation by mothers probably has a biological significance considering that flies in the wild are often under nutrient deprivation. Flies caught in the wild often have only 1–2 mature oocytes, an observation very similar to what we see under the starvation condition [28]. We believe that periodic starvation is the norm and *Drosophila* probably evolved mechanisms to ensure their reproductive capacity under such harsh conditions. Our study provides a plausible mechanism on how starved mothers produce few eggs that are primed to produce offspring that can reproduce well in the same environment, and this cycle continues in the subsequent generations to ensure continuity of the species. This enables them to survive during periods or seasons of low food source until they get access to abundant food.

Although this study has identified a memory of starvation transmitted from mother to offspring by regulating the TOR pathway activity, it is still unclear how maternal starvation influences the offspring physiology or whether the effects are transgenerational. Such analyses are hampered by the difficulty in collecting sufficient offspring from starved mothers as the rate of stage 14 oocyte production drops dramatically during starvation. In addition, only ~70% of the offspring develop into adults. Future studies will need to overcome such limitations to perform genome-wide and more comprehensive studies.

Previous studies had shown that sisRNAs and *sisR-2* are upregulated during starvation in yeast and flies, respectively [13,19–21]. In yeast, these sisRNAs consist of excised introns and introns retained in unspliced RNA. In this study, starvation promotes the expression of *sisR-8* that contains an intronic sequence retained in the mature sisRNA by alternative splicing. On the other hand, *sisR-1* to *sisR-4* are all excised introns in either linear or circular conformations [12,13,15,16]. Thus, consistent with studies performed in yeast, starvation can induce a wide variety of sisRNAs formed by different biogenesis pathways in *Drosophila*. It will be interesting to examine if such a phenomenon is conserved in mammals as well.

The molecular mechanism of how *sisR-8* regulates the splicing of its cognate gene is unclear. Since the splicing of RpL10Ab and the production of *sisR-8* are regulated by RpL10Ab protein, it is tempting to speculate that *sisR-8* may regulate the activity of RpL10Ab protein to control splicing. Repression of splicing by RpL10Ab protein may lead to production of *sisR-8*, which in turn activates local recruitment of RpL10Ab protein via a positive feedback mechanism.

Equally mysterious is how other sisRNAs *sisR-1* to *sisR-4* regulate stage 14 oocyte production during starvation. So far, few long noncoding RNAs have been implicated in regulating cell death directly. During starvation, activation of autophagy results in apoptosis of germline cells in *Drosophila* [25]. A recent study uncovered a role for vault RNAs in the regulation of autophagy in mammalian cells via the direct regulation of p62 oligomerization [29]. It will be interesting to explore if sisRNAs also regulate the autophagy pathway in future.

In summary, we propose that maternally deposited *sisR-8* and RpL10Ab regulate RpL10Ab expression to promote TOR pathway activity, which subsequently confer resistance to starvation-induced oocyte loss in the offspring.

## Materials and methods

### Identification of conserved genetic loci

Exon-intron structure refers to the arrangement and number of exons/introns within a specific gene. Related genes tend to have a similar number of exons/introns or conserved intron positions among closely related species. Thus, it has been used to predict orthologous protein-coding genes and long noncoding RNAs across species. All gene annotations were retrieved from FlyBase and UCSC Genome Browser. The orthologous intron of RpL10A was previously identified based on sequence conservation and used in this study [23].

### Fly strains

The following fly strains were used in this study: *y w* and *Oregon R* (used as control unless otherwise stated), *MTD-Gal4* [30], *TOR RNAi[HMS00904]* (Bloomington #33951), *sisR-1 RNAi* [15], *sisR-2 RNAi* [13], *sisR-3 RNAi* [12], *sisR-4* mutant [16], *RpL10Ab[CB02653]/TM3* [31], *RpL10Ab[c03865]/TM3*, *gig[G4010]/TM3 (*Bloomington #27142*)*, *rheb[EY08085]/TM3 (*Bloomington #16873). They were maintained in standard cornmeal medium unless otherwise stated. To prepare the cornmeal medium, 290.93g cornmeal, 254.5g dextrose, 118g brewer’s yeast, 40g agar and 150ml 10% Nipagin were mixed with deionized water to prepare 5 litres of food.

### Feeding protocol

To obtain RNA and protein from regularly fed female flies that are not used for the starvation assays, newly eclosed female flies were fed with yeast paste for 2 days before ovaries were dissected.

### Starvation protocol

Newly eclosed female flies were starved or fed immediately. Flies were placed in vials containing 1% agarose (dissolved in water) without or with yeast paste for 2 days before dissection for RNA or protein. For crossing, 2–7 days old fed males were added into the respective agarose vials without or with yeast paste and allowed to mate and lay eggs for 2 days. The flies were transferred to fresh vials daily. Eggs were transferred to regular fly food vials containing standard cornmeal medium and allowed to develop till adulthood.

### Starvation resistance of oocytes

Newly eclosed flies were starved or fed for 2 days before they were dissected, and the number of stage 14 oocytes scored. % of starvation-resistant stage 14 oocytes was calculated by dividing the number of stage 14 oocytes (starved) by the number of stage 14 oocytes (fed) to normalize for the maximum stage 14 oocyte production rate. For each experiment, at least 20 ovaries from at least 10 flies were counted.

### RNA extraction

RNA extraction was performed as described previously [15], using TRIzol (Ambion) and the Direct-zol miniprep kit (Zymo Research). To detect maternally deposited sisRNA and mRNA, stage 14 oocytes were manually isolated to avoid contamination with pre-mRNA in transcriptionally active germline cells. For experiments involving detection of pre-mRNA and mRNA for gene expression and splicing efficiency, whole ovaries were used as they reflect the steady-state levels of pre-mRNA and mRNA as described in previous studies [32,33].

### RT-PCR/qRT-PCR

RT-PCR/qRT-PCR were performed as described previously [15]. Transcript abundance was normalized against *actin5C* mRNA. RT was performed with M-MLV RT (Promega). PCR products were run on 1% agarose gel to visualize DNA. qRT-PCR was done using SYBR Fast qPCR kit master mix (2x) universal (Kapa Biosystems, USA) and on the Applied Biosystems 7900HT Fast Real-Time PCR system. Oligonucleotides used were rpl10ab sisR Fw GTACGTTGGCTAAGTCACCTGCGCA, rpl10ab sisR Rv TTGGCCAGCTTCTTCACCAGCTTCT, RpL10Ab spliced Fw TGCAGATCGGCCTGAAGAACTACGA, RpL10Ab spliced Rv CAATGCTGCTGATCGCCAAGGATGCA, RpL10Ab pre-mRNA fwp GTAAGTTTTCCATGAGGCCCCACTT, RpL10Ab pre-mRNA rvp CAGCTTCTTCACCAGCTTCTTGTTC, actin5C Fw TGCCCATCTACGAGGGTTAT, actin5C Rv AGTACTTGCGCTCTGGCGG.

### Calculation of splicing index

Splicing index was calculated as previously described [32]. qRT-PCR was used to quantify the spliced RNA using primers that flank the intron of interest. Unspliced RNA was measured by qPCR using primers that amplify pre-mRNA. Ct values for spliced and unspliced RNA were normalized against *actin5C* before the ratio was calculated. Splicing indices for controls were set to 1 for comparison. An increase in splicing index means an increase in spliced/unspliced RNA ratio, hence more efficient splicing, and vice versa.

### Transgene and plasmid construction

Cloning of sisRNA intron overexpression construct was performed as described previously [15]. Full length *sisR-8* intron was inserted into a UAS-dsRed-intron-myc plasmid [34]. Cloning of RpL10Ab CDS overexpression construct was performed as described previously using the Gateway system [35] into pPFW. Injection was done by BestGene Inc.

### *Drosophila* S2 cell culture

S2 cells that can grow in serum-free conditions were obtained from Steve Cohen’s laboratory. They were cultured at 25°C in Schneider’s medium (Invitrogen) supplemented with 2 mM glutamine without serum.

### Transfection of S2 cells

S2 cells were transfected using Cellfectin (Invitrogen) in accordance with the manufacturer’s protocol. For overexpression of *sisR-8*, 2μg of UAS-dsRed-sisR-8 intron-myc and 2μg of ub-Gal4 were co-transfected. As control, ub-Gal4 was transfected alone. For overexpression of RpL10Ab, 2μg pAFW-RpL10Ab CDS was transfected and non-transfected cells were used as control. Cells were harvested 48 hr after transfection.

### Western blot

Western blotting was done as described previously [16]. S2 cells and dissected ovaries were homogenized in 2X sample buffer containing beta-mercaptoethanol. Transfer of TSC2 (a large protein) was performed at 10V for 16 hours. Primary antibodies used were rabbit anti-pS6K (1:1,000, Cell Signaling #9209), rabbit anti-pAKT (1:1,000, Cell Signaling #4054), rabbit anti-AKT (1:1,000, Cell Signaling #9272), guinea pig anti-S6K (1:3,000, gift from Aurelio Teleman) [36], mouse anti-Gig/TSC2 (1:500, DSHB 3H1D8F5), rabbit anti-RpL10A (1:2,000, abcam ab187998) and mouse anti-Actin (1:100, DSHB JLA20). Coomassie blue staining or immunoblotting for Actin was done to determine equivalent loading. Detection was done using the ChemiDoc Touch Imaging System (BioRad) under non-saturating conditions. Quantification of western blots was performed using imageJ software as previously described [16].

### Immunoprecipitation

Immunoprecipitation was done as described previously [37]. S2 cells were lysed in protein extraction buffer (50mM Tris-HCl pH 7.5, 150mN NaCl, 5mM MgCl_2_, 0.1% NP-40) supplemented with Protease Inhibitor Cocktail (Roche). This was followed by blocking the cell lysates with protein A/G agarose beads (Merck Millipore). Antibodies (anti-RpL10A or anti-Gig) were added and incubated overnight at 4°C. For negative controls, no antibody was added. The next day, protein A/G beads were added and incubated for 2 hr before washing in protein extraction buffer 3 times. Thereafter, protein and RNA were extracted using 2X sample buffer containing beta-mercaptoethanol and Direct-zol RNA miniprep kit (Zymo Research), respectively.

### Immunostaining

Immunostaining was performed as described previously [35,38]. Briefly, ovaries were fixed in 16% PFA: Grace’s media (ratio 1:2) for 10–20 min before washing with PBS-T (0.2% Triton) and blocked for 30 min in 5% normal goat serum. Ovaries were incubated in primary antibody mix at room temperature overnight. The next day, they were washed in PBS-T and incubated in secondary antibody mix for 4 hours at room temperature. DNA was stained using DAPI and ovaries were mounted in Vectashield and examined under the Leica SPEII microscope. Primary antibody used was rabbit anti-cleaved *Drosophila* Dcp-1 (Asp216) antibody (Cell Signaling #9578), guinea pig anti-Vasa antibody [39], rabbit anti-pS6K (1:200, Cell Signaling #9209) and rabbit anti-pAKT (1:200, Cell Signaling #4054). The percentage of apoptotic stage 8/9 egg chambers was calculated by scoring the number of egg chambers positive for Dcp-1 over the total number of stage 8/9 egg chambers from 6–8 ovaries. Quantification of signal intensities was performed using identical confocal settings and imageJ software as previously described [21].

### Statistics

For all experiments, the tests and the number of independent biological replicates were indicated in the figure legends or figures. P values and definitions of error bars were indicated in the legends. Sample sizes were not pre-determined prior to the experiments. T-tests were performed on samples that are normally distributed.

## Supporting information

S1 Fig PEPCK expression in starved and TOR RNAi ovaries. (a) Fold-change of PEPCK in stage 14 oocytes from fed and starved F0 mothers. (b) Fold-change of PEPCK in *MTD-Gal4>TOR* RNAi compared to control ovaries. Error bars represent sd from mean from 3 biological replicates. All qRT-PCR were normalized against *actin5C* mRNA.
(TIF)

S2 Fig Paternal *RpL10Ab* is not required for transmission of starvation memory. (a) Crossing scheme to reduce a copy of *RpL10Ab* gene in the F0 fathers and the collection of *TM/+* F1 offspring. (b) Fold-change of PEPCK in fed F1 offspring ovaries from *RpL10Ab[CB2653]/+* F0 fathers compared to those from wildtype F0 fathers. Error bars represent sd from mean from 3–4 biological replicates. **: p<0.01. (c) Fold-change of RpL10Ab mRNA in fed F1 offspring ovaries from *RpL10Ab[CB2653]/+* F0 fathers compared to those from wildtype F0 fathers. Error bars represent sd from mean from 3–4 biological replicates. **: p<0.01. All qRT-PCR were normalized against *actin5C* mRNA.
(TIF)

S3 Fig Characterization of *RpL10Ab[CB2653]/+*, *RpL10Ab[C03865]/+* and RpL10Ab OE ovaries. (a, b) Fold-change of PEPCK and 4E-BP in fed controls versus *RpL10Ab[CB2653]/+* ovaries. **: p<0.01, t-test. Error bars depict SD from n = 3–5 biological replicates.(C, D) Fold-change of RpL10Ab mRNA and PEPCK in fed *RpL10Ab[C03865]/+* ovaries compared to control ovaries. *: p<0.05, t-test. Error bars represent sd from mean from 3 biological replicates.(E) Fold-change of PEPCK in fed controls versus *MTD-Gal4>FLAG-RpL10Ab* ovaries. **: p<0.01, t-test. Error bars depict SD from n = 3 biological replicates. All qRT-PCR were normalized against *actin5C* mRNA.
(TIF)

S4 Fig Staining of pAKT and pS6K in *RpL10Ab/+* mutant and MTD>RpL10Ab OE ovaries. (a) Confocal images showing the levels of pAKT and pS6K, together with Vasa, in egg chambers from fed *Oregon R* and *RpL10Ab[CB02653]/+* F0 flies. (b) Quantification of the pAKT and pS6K signal intensities (normalized to Vasa) shown in (a). Error bars depict SD from n = 8 egg chambers from 6 ovaries. **p<0.01. (c) Confocal images showing the levels of pAKT and pS6K, together with Vasa, in egg chambers from fed *Oregon R* and *MTD>RpL10Ab OE* F0 flies. (d) Quantification of the pAKT and pS6K signal intensities in both germline and somatic follicle cells (normalized to Vasa) shown in (c). Down-regulation of pAKT and pS6K signals was seen in the germline cells but not in the somatic follicle cells due to the overexpression of RpL10Ab driven by MTD-Gal4 in the germline cells. Error bars depict SD from n = 6 egg chambers from 6 ovaries. **p<0.01.
(TIF)

S5 Fig Percentage of apoptotic stage 8/9 egg chambers in fed and starved *Oregon R*, *RpL10Ab/+* and *gig/+* ovaries. (a) Confocal images showing examples of apoptotic stage 8/9 egg chambers (arrowheads) of the indicated genotypes stained positive for cleaved *Drosophila* Dcp-1 (green). DAPI in blue. (b) % of apoptotic stage 8/9 egg chambers are significantly reduced in starved *RpL10Ab[CB2653]/+* and starved *gig[G4010]/+* ovaries compared to starved *Oregon R*. **: p<0.01, t-test. Error bars depict SD from n = 3 biological replicates.
(TIF)
