# Supplementary figures and images for: Maternal starvation primes progeny response to nutritional stress
Source: PLoS Genet. 2021 Nov 29;17(11):e1009932. doi: 10.1371/journal.pgen.1009932 (PMC8659306; doi:10.1371/journal.pgen.1009932)

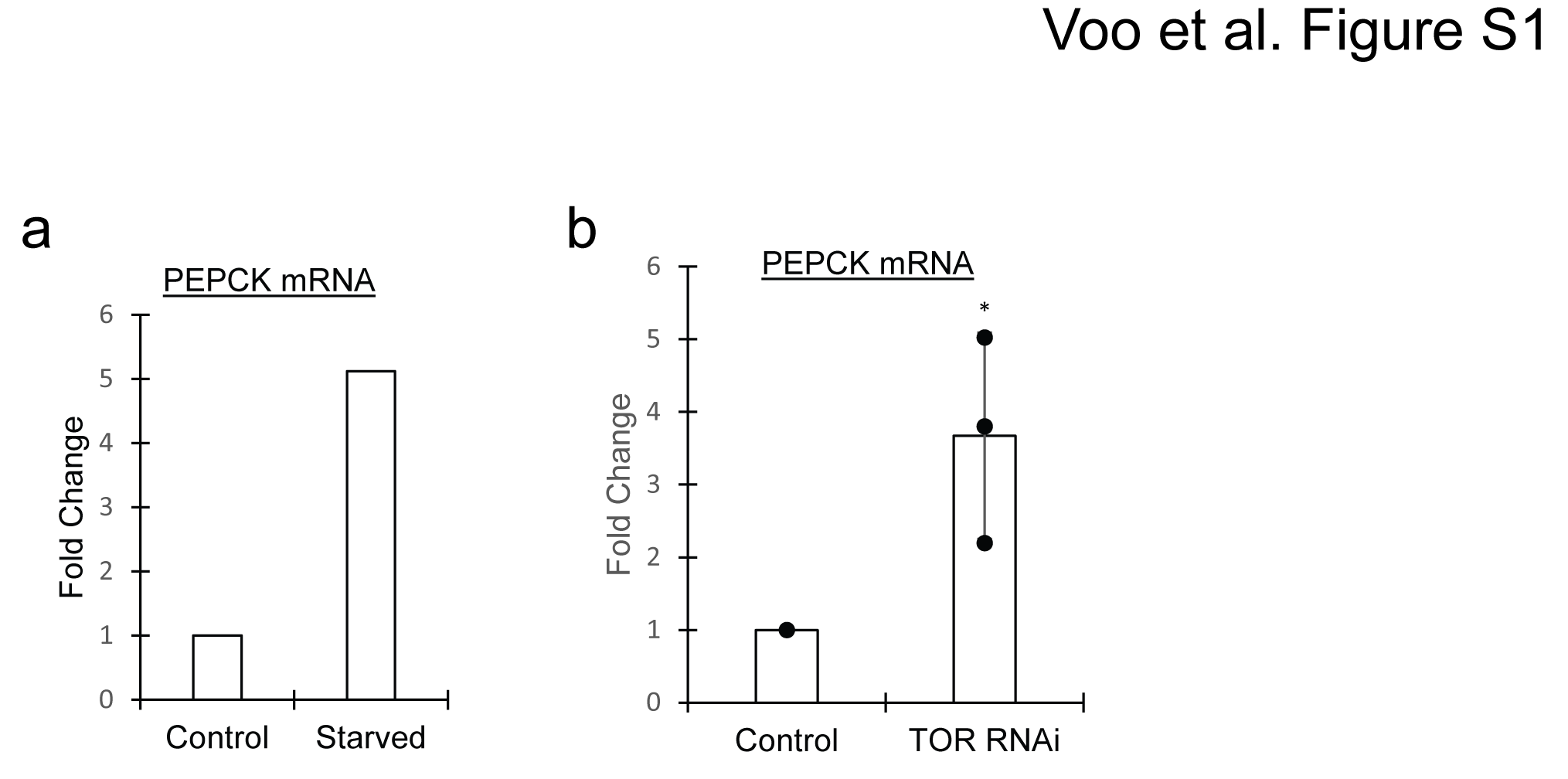

Supplement: S1 Fig — (a) Fold-change of PEPCK in stage 14 oocytes from fed and starved F0 mothers. (b) Fold-change of PEPCK in MTD-Gal4>TOR RNAi compared to control ovaries. Error bars represent sd from mean from 3 biological replicates. All qRT-PCR were normalized against actin5C mRNA. (TIF) [file pgen.1009932.s001.tif]

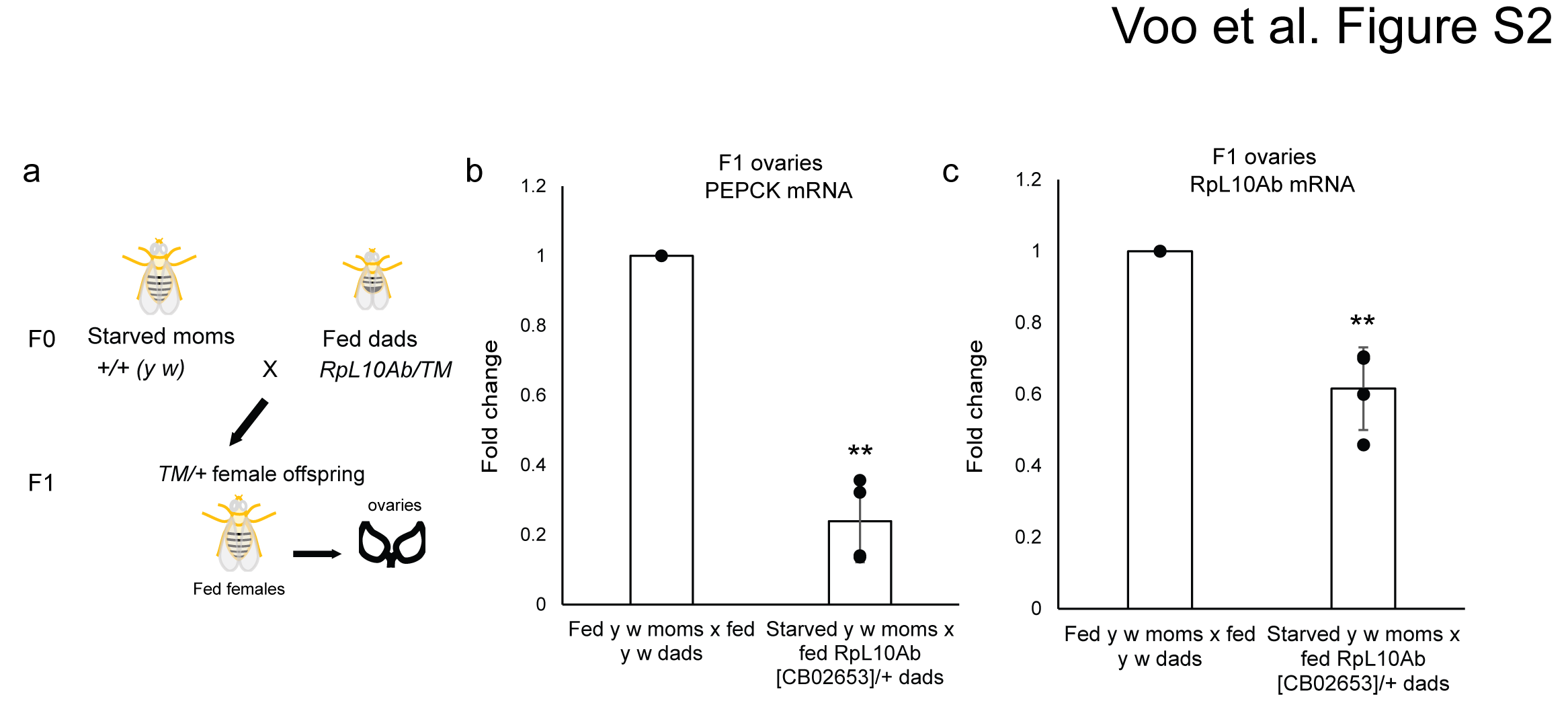

Supplement: S2 Fig — (a) Crossing scheme to reduce a copy of RpL10Ab gene in the F0 fathers and the collection of TM/+ F1 offspring. (b) Fold-change of PEPCK in fed F1 offspring ovaries from RpL10Ab[CB2653]/+ F0 fathers compared to those from wildtype F0 fathers. Error bars represent sd from mean from 3–4 biological replicates. **: p<0.01. (c) Fold-change of RpL10Ab mRNA in fed F1 offspring ovaries from RpL10Ab[CB2653]/+ F0 fathers compared to those from wildtype F0 fathers. Error bars represent sd from mean from 3–4 biological replicates. **: p<0.01. All qRT-PCR were normalized against actin5C mRNA. (TIF) [file pgen.1009932.s002.tif]

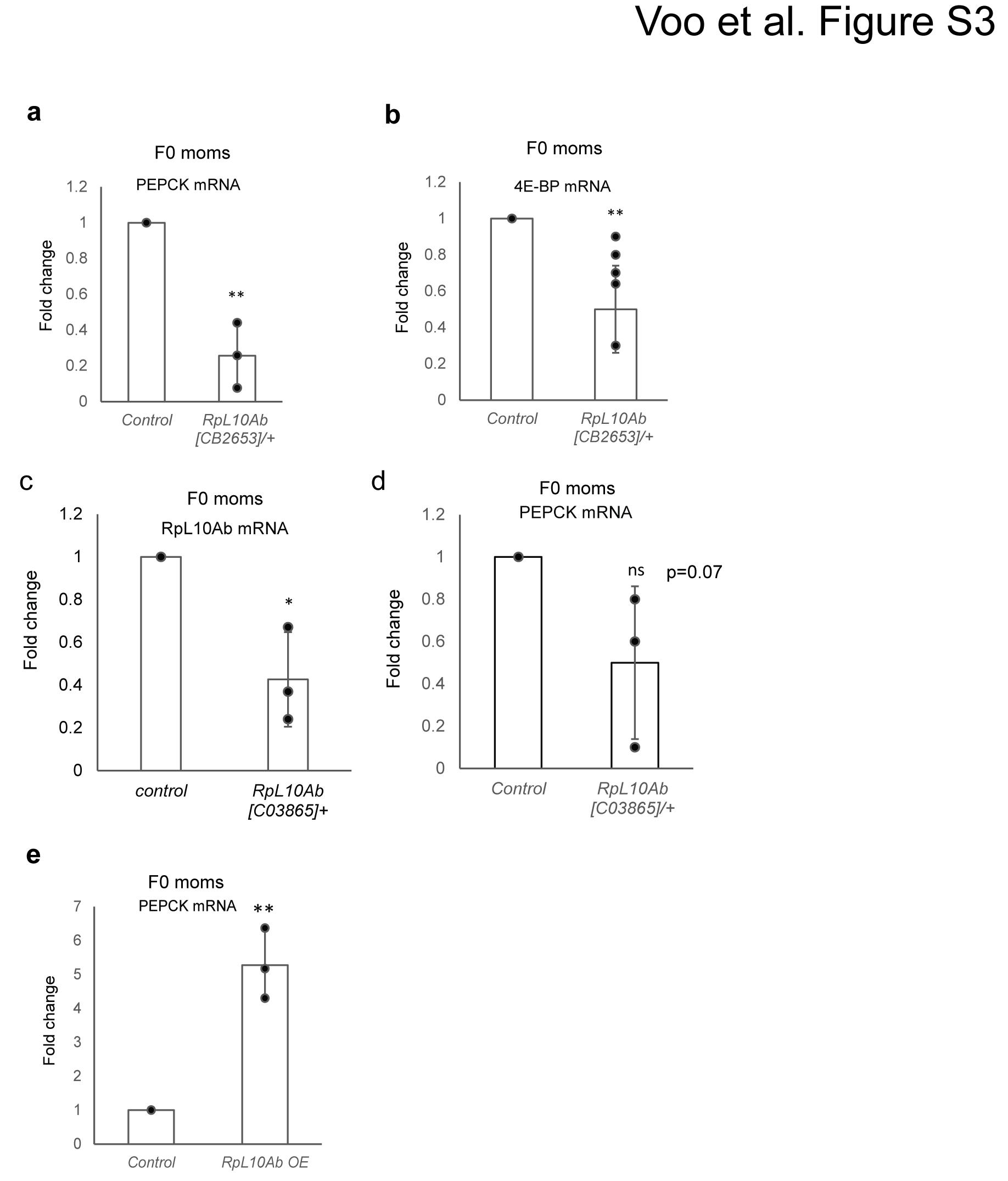

Supplement: S3 Fig — (a, b) Fold-change of PEPCK and 4E-BP in fed controls versus RpL10Ab[CB2653]/+ ovaries. **: p<0.01, t-test. Error bars depict SD from n = 3–5 biological replicates.(C, D) Fold-change of RpL10Ab mRNA and PEPCK in fed RpL10Ab[C03865]/+ ovaries compared to control ovaries. *: p<0.05, t-test. Error bars represent sd from mean from 3 biological replicates.(E) Fold-change of PEPCK in fed controls versus MTD-Gal4>FLAG-RpL10Ab ovaries. **: p<0.01, t-test. Error bars depict SD from n = 3 biological replicates. All qRT-PCR were normalized against actin5C mRNA. (TIF) [file pgen.1009932.s003.tif]

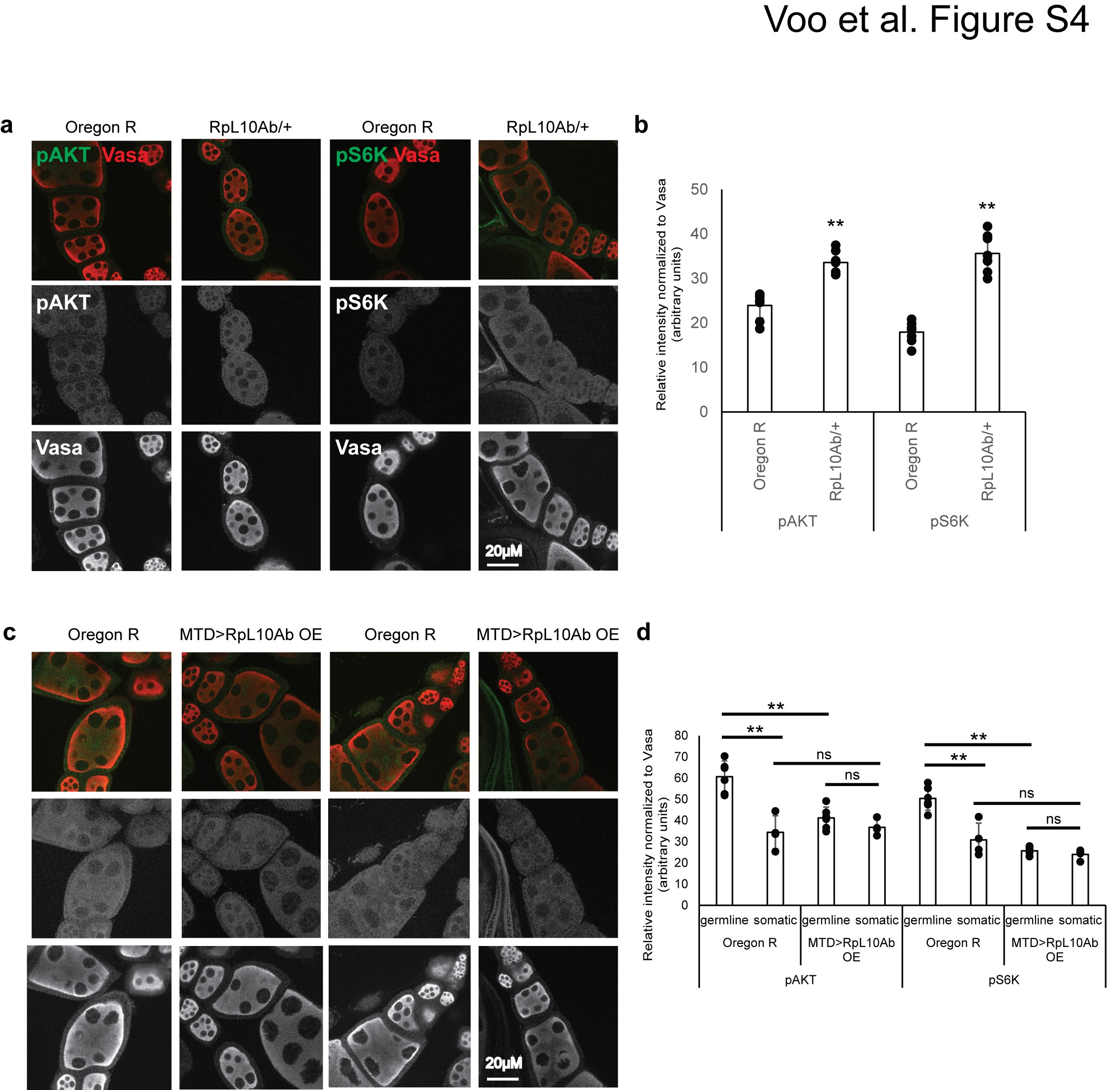

Supplement: S4 Fig — (a) Confocal images showing the levels of pAKT and pS6K, together with Vasa, in egg chambers from fed Oregon R and RpL10Ab[CB02653]/+ F0 flies. (b) Quantification of the pAKT and pS6K signal intensities (normalized to Vasa) shown in (a). Error bars depict SD from n = 8 egg chambers from 6 ovaries. **p<0.01. (c) Confocal images showing the levels of pAKT and pS6K, together with Vasa, in egg chambers from fed Oregon R and MTD>RpL10Ab OE F0 flies. (d) Quantification of the pAKT and pS6K signal intensities in both germline and somatic follicle cells (normalized to Vasa) shown in (c). Down-regulation of pAKT and pS6K signals was seen in the germline cells but not in the somatic follicle cells due to the overexpression of RpL10Ab driven by MTD-Gal4 in the germline cells. Error bars depict SD from n = 6 egg chambers from 6 ovaries. **p<0.01. (TIF) [file pgen.1009932.s004.tif]

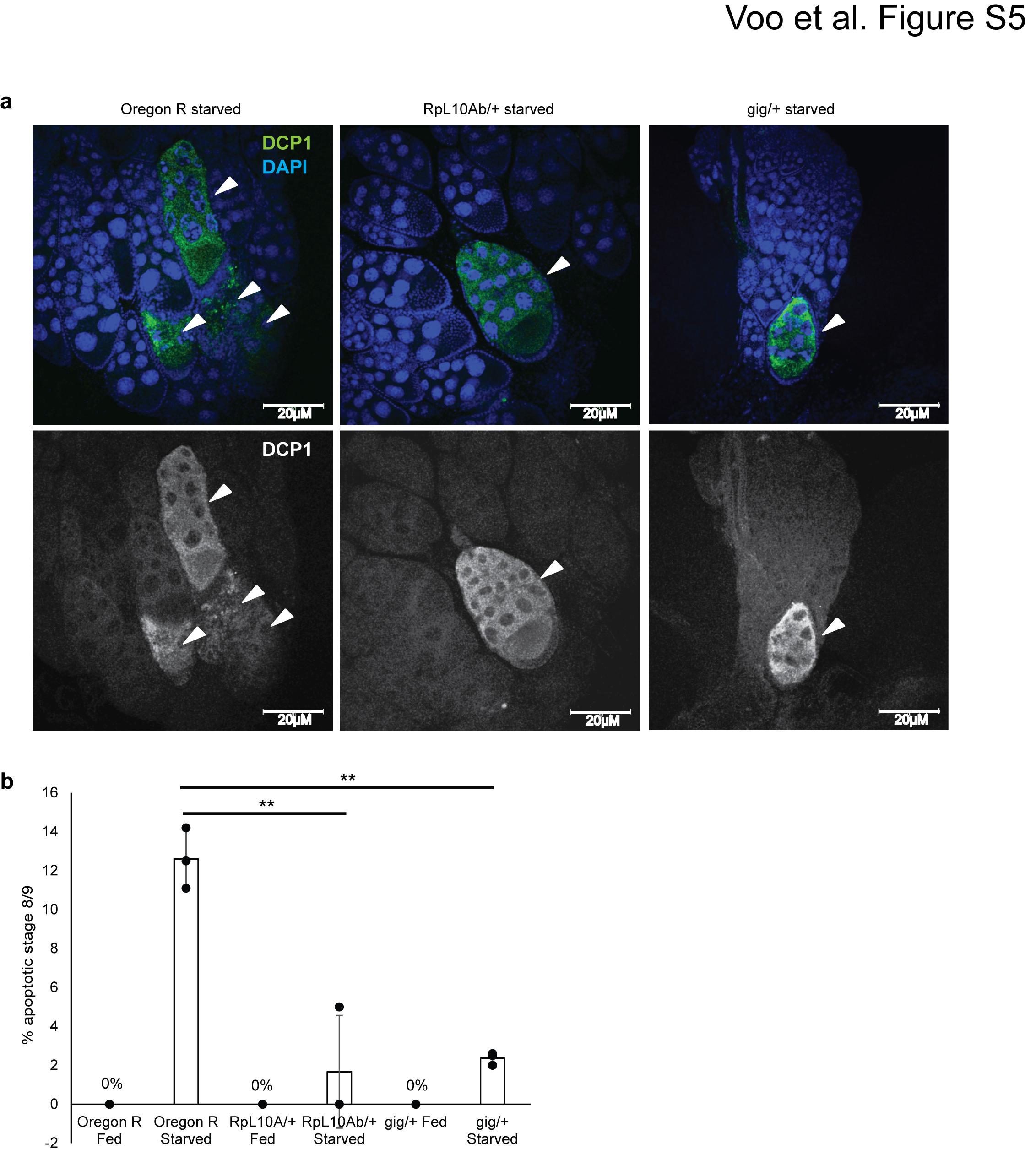

Supplement: S5 Fig — (a) Confocal images showing examples of apoptotic stage 8/9 egg chambers (arrowheads) of the indicated genotypes stained positive for cleaved Drosophila Dcp-1 (green). DAPI in blue. (b) % of apoptotic stage 8/9 egg chambers are significantly reduced in starved RpL10Ab[CB2653]/+ and starved gig[G4010]/+ ovaries compared to starved Oregon R. **: p<0.01, t-test. Error bars depict SD from n = 3 biological replicates. (TIF) [file pgen.1009932.s005.tif]
